# Supplementary material for: Identification of photoperiod-induced specific miRNAs in the adrenal glands of Sunite sheep (Ovis aries)
Source: Front Vet Sci. 2022 Jul 22;9:888207. doi: 10.3389/fvets.2022.888207 (PMC9354845; doi:10.3389/fvets.2022.888207)
Supplement: Supplementary file 1 [file Data_Sheet_1.ZIP › Supplementary file/miRDeep2 file.zip!/miRDeep2 file/Know_Structure/SP42A2/oar-mir-143.pdf]

miRBase precursor : oar-mir-143
Total read count : 515499
oar-miR-143 read count : 511987
remaining reads : 3512

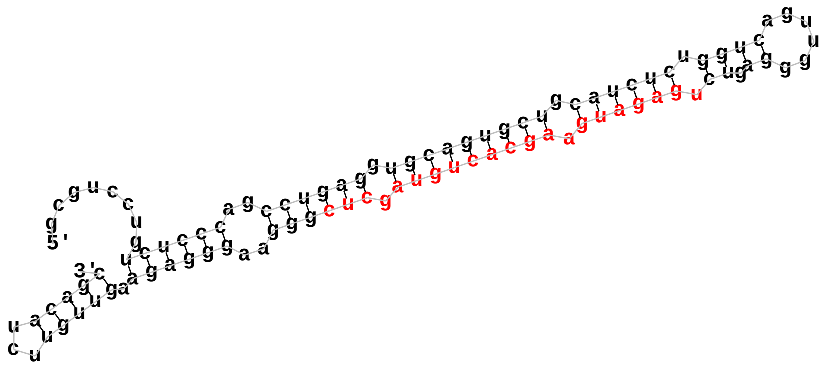

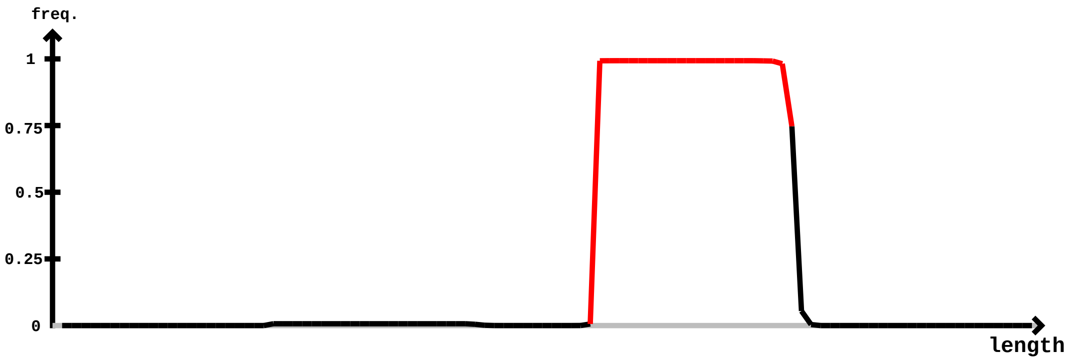

oar-miR-143

```
5'- gcguccugucucccagccugaggugcagugcugcaucucuggucaguugggagucugagaugaagcacuguagcucgggaagggagaaguuguucuacagc  -3'    exp
    ........((((((..((((((.(((((((((.((((((.((((......)).)).)))))).))))))))).))))))..)))))).(((((...)))))   reads      mm         sample
    ....................aggugcagugcugcaucucug............................................................   1          0          seq
    ....................aggugcagugcugcaucucugg...........................................................   1          0          seq
    ....................aggugcagugcugcaucucuggu..........................................................   1          0          seq
    .....................ggugcagugcugcaucucu.............................................................   9          0          seq
    .....................ggugcagugcugcaucucug............................................................   13         0          seq
    .....................ggugcagugcugcaucucugg...........................................................   984        0          seq
    .....................ggugcagugcugcaucucuggu..........................................................   1884       0          seq
    .....................ggugcagugcugcaucucugguc.........................................................   562        0          seq
    .....................ggugcagugcugcaucucuggguca........................................................   50         0          seq
    .....................ggugcagugcugcaucucuggucagu......................................................   1          0          seq
    ......................gugcagugcugcaucucugg...........................................................   1          0          seq
    ......................gugcagugcugcaucucugguc.........................................................   1          0          seq
    .......................ugcagugcugcaucucuggucaguu.....................................................   1          0          seq
    ............................................aguugggagucugagaugaagcacuguagcuc.........................   2          0          seq
    ...............................................ugggagucugagaugaagcacu................................   1          0          seq
    .....................................................ucugagaugaagcacugu..............................   1          0          seq
    .....................................................ucugagaugaagcacuguag............................   2          0          seq
    .....................................................ucugagaugaagcacuguagc...........................   12         0          seq
    .....................................................ucugagaugaagcacuguagcu..........................   7          0          seq
    .....................................................ucugagaugaagcacuguagcuc.........................   53         0          seq
    ......................................................cugagaugaagcacugu..............................   8          0          seq
    ......................................................cugagaugaagcacuguag............................   2          0          seq
    ......................................................cugagaugaagcacuguagc...........................   68         0          seq
    ......................................................cugagaugaagcacuguagcu..........................   732        0          seq
    ......................................................cugagaugaagcacuguagcuc.........................   2175       0          seq
    ......................................................cugagaugaagcacuguagcucg........................   53         0          seq
    ......................................................cugagaugaagcacuguagcucgg.......................   1          0          seq
    .......................................................ugagaugaagUacugua.............................   5          1          seq
    .......................................................ugagaugaagcacugua.............................   274        0          seq
    .......................................................ugaCaugaagcacugua.............................   7          1          seq
    .......................................................ugagaugaagcacuguU.............................   3          1          seq
    .......................................................ugagaugaagcacuguG.............................   4          1          seq
    .......................................................ugagaugaagcGcugua.............................   1          1          seq
    .......................................................ugagaugGagcacugua.............................   3          1          seq
    .......................................................ugagGugaagcacugua.............................   8          1          seq
```

oar-miR-143

```
gcguccugucucccagccugaggugcagugcugcaucucuggucaguugggagucugagaugaagcacuguagcucgggaagggagaaguuguucuacagc
.......................................................ugagaugaagcacugAa.............................   1        1      seq
.......................................................ugagauAaagcacuguag............................   2        1      seq
.......................................................ugagaugaagcacuguag............................   391      0      seq
.......................................................ugagaugaagcacuguagc...........................   5141     0      seq
.......................................................ugagaugaagcacuguagcu..........................   120266   0      seq
.......................................................ugagaugaagcacuguagcuc.........................   354583   0      seq
.......................................................ugagaugaagcacuguagcucg........................   26049    0      seq
.......................................................ugagaugaagcacuguagcucgg.......................   1930     0      seq
.......................................................ugagaugaagcacuguagcucggg......................   5        0      seq
........................................................gagaugaagcacuguagc...........................   4        0      seq
........................................................gagaugaagcacuguagcu..........................   27       0      seq
........................................................gagaugaagcacuguagcuc.........................   115      0      seq
........................................................gagaugaagcacuguagcucg........................   10       0      seq
.........................................................agaugaagcacuguagcu..........................   2        0      seq
.........................................................agaugaagcacuguagcucg........................   1        0      seq
.........................................................agaugaagcacuguagcucgg.......................   20       0      seq
.........................................................agaugaagcacuguagcucggg......................   4        0      seq
..........................................................gaugaagcacuguagcuc.........................   1        0      seq
..........................................................gaugaagcacuguagcucgg.......................   10       0      seq
..........................................................gaugaagcacuguagcucggg......................   1        0      seq
..........................................................gaugaagcacuguagcucggga.....................   3        0      seq
..........................................................gaugaagcacuguagcucgggaa....................   1        0      seq
............................................................ugaagcacuguagcucg........................   1        0      seq
```
